# Protective Role of PGC-1α in Diabetic Nephropathy Is Associated with the Inhibition of ROS through Mitochondrial Dynamic Remodeling

**DOI:** 10.1371/journal.pone.0125176

**Published:** 2015-04-08

**Authors:** Kaifeng Guo, Junxi Lu, Yan Huang, Mian Wu, Lei Zhang, Haoyong Yu, Mingliang Zhang, Yuqian Bao, John Cijiang He, Haibing Chen, Weiping Jia

**Affiliations:** 1 Department of Endocrinology and Metabolism, Shanghai Clinical Center for Diabetes, Shanghai Diabetes Institute, Shanghai Key Laboratory of Diabetes Mellitus, Shanghai Jiaotong University Affiliated Sixth People’s Hospital, Shanghai, 200233, China; 2 Division of Nephrology, Department of Medicine, Icahn School of Medicine at Mount Sinai, New York, NY, United States of America; 3 Department of Pharmacology and Systems Therapeutics, Icahn School of Medicine at Mount Sinai, New York, NY, United States of America; University of Illinois at Chicago, UNITED STATES

## Abstract

The overproduction of mitochondrial reactive oxygen species (ROS) plays a key role in the pathogenesis of diabetic nephropathy (DN). However, the underlying molecular mechanism remains unclear. Our aim was to investigate the role of PGC-1α in the pathogenesis of DN. Rat glomerular mesangial cells (RMCs) were incubated in normal or high glucose medium with or without the PGC-1α-overexpressing plasmid (pcDNA3-PGC-1α) for 48 h. In the diabetic rats, decreased PGC-1α expression was associated with increased mitochondrial ROS generation in the renal cortex, increased proteinuria, glomerular hypertrophy, and higher glomerular 8-OHdG (a biomarker for oxidative stress). In vitro, hyperglycemia induced the downregulation of PGC-1α, which led to increased DRP1 expression, increased mitochondrial fragmentation and damaged network structure. This was associated with an increase in ROS generation and mesangial cell hypertrophy. These pathological changes were reversed in vitro by the transfection of pcDNA3-PGC-1α. These data suggest that PGC-1α may protect DN via the inhibition of DRP1-mediated mitochondrial dynamic remodeling and ROS production. These findings may assist the development of novel therapeutic strategies for patients with DN.

## Introduction

Diabetic nephropathy (DN) is one of the major complications of diabetes and leads to end-stage renal disease. The common pathological features of DN are mesangial cell proliferation, glomerular hypertrophy, and thickening of the glomerular basement membrane [1] [2], which ultimately result in fibrosis and chronic renal failure. However, it is known that early metabolic changes, such as deficient oxygen handling, diminished microcirculation, and mitochondrial dysfunction, precede these structural changes [3–5]. Specifically, mitochondrial dysfunction and the overproduction of mitochondrial reactive oxygen species (ROS) play an important role in the pathogenesis of DN [6,7]. Using cultured aortic endothelial cells, it was found that hyperglycemia leads to the overproduction of mitochondrial ROS, which has been proposed to serve as the common link for several key pathogenic pathways involved in the microvascular complications of diabetes, including DN [8]. However, the specific signaling molecules that couple hyperglycemia with mitochondrial ROS production and the nature of mitochondrial dysfunction in the diabetic milieu remain unidentified.

It is well known that metabolic homeostasis is to a large extent controlled by transcriptional regulatory mechanisms, which involve the coordinated action of transcription factors, cofactors and the transcription initiation machinery that regulates the expression of genes involved in mitochondrial biogenesis and function [9]. In patients with diabetes, this metabolic adaptation occurs at a cellular level, and the fine-tuning of mitochondrial function is mainly under the control of peroxisome proliferator-activated receptor-γ coactivator-1α (PGC-1α) [1,10,11]. PGC-1α, which is sensitive to energy metabolism, is a major regulator of oxidative metabolism and mitochondrial biogenesis. As a positive regulator of oxidative metabolism, PGC-1α co-regulates a set of proteins that participate in the cellular response to mitochondrial oxidative stresses, such as hyperglycemia [12]. Our previous study demonstrated that PGC-1α regulates the expression of vascular endothelial growth factor (VEGF) induced by hyperglycemia through the inhibition of mitochondrial ROS in cultured bovine retinal capillary endothelial cells [13]; however, little is known about its role in rat glomerular mesangial cells (RMCs) and kidney tissue.

Thus, the aim of this study was to investigate whether PGC-1α is involved in the pathogenesis of DN. Our results suggest that the overproduction of mitochondrial ROS was associated with decrease expression levels of PGC-1α in the kidneys of diabetic rats. The overexpression of PGC-1α improves the function of glomerular mesangial cells under hyperglycemic conditions. This finding was associated with the inhibition of ROS production via the regulation of DRP1-mediated mitochondrial dynamic remodeling.

## Methods

The study was approved by the animal care committee of Shanghai Jiao Tong University Affiliated Sixth People’s Hospital and complied with the Institutional Animal Care and Use Committee (IACUC).All chemicals were of reagent-grade quality and were purchased from Sigma Chemicals (St. Louis, MO), unless stated otherwise.

### Animal studies

Male Sprague-Dawley (SD, 8 weeks old) rats were obtained from Shanghai SiLaike (SLAC) Laboratory Animal Company (Shanghai, China). The animals were housed individually in stainless-steel wire-bottomed cages in an air-conditioned room under the following conditions: temperature, 22°C ± 2°C; relative humidity, 50% ± 10%; and a 12:12 h light: dark cycle. The animals had free access to commercial pelleted rat food and water. Ten rats were fed with chow as control group. Another twenty rats were fed with high fat diet. After one month, those ten SD rats fed with high fat diet were administered an intraperitoneal injection of streptozotocin (STZ, 30 mg/kg body weight; Sigma, St. Louis, MO) in 0.05 mol/l citrate buffer (pH 4.4) to induce diabetes mellitus (DM). Blood glucose levels were determined using a glucose analyzer 2 days after the injection, and rats with glucose levels greater than 16.7 mmol/l were considered to have diabetes. After 12 weeks, 24-h urine was collected from each rat in individual metabolic cage and centrifuged at 2,000g for 5 min and the rats were sacrificed. Blood samples were collected and serum was prepared, and tissues were immediately excised. Samples were collected at the same time from a group of control rats, which did not receive the STZ injection and were therefore non-diabetic. 24h microalbuminuria was measured by Dade Behring Nephelometer II System (antiserum to albumin, Siemens Healthcare Diagnostics). Urine albumin excretion (UAE) was expressed as milligrams albumin in 24-h urine. The levels of glucose, cholesterol, triglycerides, and other indicators were determined in the blood samples, and the tissues were subject to histological examination.

### Histological examination of renal tissue

A slice of kidney tissue was fixed in 10% formalin for 1 week at 4°C. Then, the specimens were dehydrated in a graded series of ethanol, cleared in xylene, and embedded in paraffin wax. Tissue blocks were cut into 5-μm thick sections using a rotary microtome, and the sections were stained using hematoxylin-eosin. For immunohistochemical analysis, the sections were deparaffinized, endogenous peroxidase activity was quenched in 3% H2O2 for 10min, the sections were washed in PBS, incubated with LCA-Biotin (Vector Labs) or anti-FN- antibody (EPITOMICS) for 1 h, and then incubated with the appropriate biotinylated secondary antibodies, followed by treatment with avidin—biotin coupling (ABC) reagent (Vector Laboratories) as recommended by the manufacturer. Color development was achieved with the substrate diaminobenzidine. Slides were then counterstained with Mayer’s hematoxylin and mounted in glycerol jelly. The stained sections were viewed under a light microscope by a pathologist without prior knowledge of the groups. Quantification of positive areas of immunostaining for FN antibody (brown color) in the glomerular was evaluated by computer-based morphometric analysis using the NIH-developed Image J software (Wayne Rasband, NIH).

### Immunofluorescence staining for 8-OHdG

8-Hydroxy-2'-deoxyguanosine (8-OhdG) is a mutation-prone (G:C to T:A tranversion) DNA base-modified product generated by reactive oxygen species or photodynamic action. It is one of the most commonly used markers for the evaluation of oxidative DNA damage. Immunofluorescence staining for 8-oxo-2′-deoxyguanosine (8-oxo-dG) was carried out using anti-8-oxo-dG antibody (Trevigen, Gaithersburg, MD, USA) and visualized with AlexaFluor 488-nm antibody (Invitrogen). The samples were analyzed by using an inverted fluorescence microscope equipped with a CCD camera (ZEISS Axiovert 40 CFL), image processing was performed using the ZEISS AxioVision software (Carl Zeiss AG) and the intensity of green fluorescence was measured by using the NIH-developed ImageJ software (Wayne Rasband, NIH).

### Measurement of ROS

Renal cortex mitochondria ROS production was detected as described by Benani et al. [14]. Renal issues were harvested in cold buffered medium (5 mmol/l HEPES in PBS) and immediately frozen in liquid nitrogen to improve the following probe diffusion. After rapid thawing, medium was discarded. Samples were exposed to 8 μmol/l Mitochondrial ROS dissolved in 400 μl fresh medium and were incubated at 37°C for 30 min under agitation. Medium was then removed, and samples were further incubated in a lysis buffer (0.1% SDS, Tris-HCl, pH 7.4) for 15 min at 4°C. After homogenization, samples were centrifuged at 6,000g for 20 min at 4°C. Supernatants were collected and subjected to fluorescence analysis as stated previously.

Mitochondrial ROS generation was assessed using MitoSOX Red (Invitrogen/Molecular Probes), which is a fluorogenic dye that is taken up by mitochondria, where it is readily oxidized by superoxide, but not by other ROS. Cells were loaded with 1μM MitoSOX Red in phenol-free MEM for 10 min at 37°C, then, washed with warm buffer. MitoSOX Red fluorescent intensity was determined at 515-nm excitation and 580-nm emission. The brightness intensity of MitoSOX signal was semiquantified by using NIH Image J software. The data shown represent three separate experiments, and are expressed as relative fluorescence intensity.

### Cell culture and transfection experiments

RMCs were routinely cultured in minimum essential medium (MEM) (Invitrogen) containing 5.6 mM glucose that was supplemented with 10% fetal bovine serum and 1% penicillin/streptomycin (Invitrogen). The cells were grown at 37°C in a 5% CO_2_ atmosphere. At approximately 70% confluence, the cells were subcultured in fresh MEM. The cells were then made quiescent by incubation in serum-free medium for 24 h. For determination of the effects of high glucose levels on PGC-1α expression and the sustained effect of high glucose on mitochondrial morphology, RMCs were grown in 5.6 mM normal glucose (NG) or 30 mM high d-glucose (HG) medium for 48 h. RMCs grown in 5.6 mM NG medium that contained 24.4 mM mannitol (Man) were used as the osmotic control. The expression plasmid pcDNA3-PGC-1α encoding PGC-1α was constructed by our laboratory and plasmid expressing shRNA for PGC-1α, DRP1 and its control shRNA was purchased from Thermo Scientific. Cells were transiently transfected with plasmid for 24 h before the experiments, using Lipofectamine 2000 Reagent (Invitrogen) according to the manufacturer’s recommendations. The incubation concentration of DRP1 recombinant protein (OriGene Technologies Inc) was 10ng/ml.

### Mitochondrial Morphology labeling and analysis

For morphometric analyses of mitochondria, digital images were acquired for mitochondria labeled by immunofluorescence using the MitoTracker stain (MitoTracker Red CMX Ros; Invitrogen), which passively diffuses across the plasma membrane and accumulates in active mitochondria. After plasmid treatment, RMCs were incubated in growth medium containing MitoTracker stock solution (1 mM) diluted to a final working concentration (100 nM). When cells reached the desired confluence, the medium was removed from the dish, and pre-warmed (37°C) staining solution containing the MitoTracker probe was added; the medium was then incubated for 25 min under growth conditions. After staining, the staining solution was replaced with fresh pre-warmed media and the cells were observed under a Zeiss fluorescence microscope (Zeiss, Jena, Germany). Quantitative analysis of mitochondrial morphology was conducted using computer-assisted morphometric analysis for the calculation of form factor (FF) and aspect ratio (AR) values [15,16]. Digital images were processed through a convolve filter using the NIH-developed Image J software to obtain isolated and equalized fluorescence pixels. Mitochondria were subjected to particle analysis for acquiring FF values (4π area/perimeter^2^) and AR values, which are derived from the lengths of major and minor axes. An AR value of 1 indicates a perfect circle, and as mitochondria elongate and become more elliptical, AR increases. An FF value of 1 corresponds to a circular, unbranched mitochondrion, and higher FF values indicate a longer, more branched mitochondrion. Detailed analysis step was presented in the S1 Fig.

### Total amount of protein/cell number ratio

Because the total amount of protein/cell number ratio is a well-established measure of cellular hypertrophy, this parameter was used to determine whether the alteration of cell growth was accompanied by cell hypertrophy [17]. After plasmid transfection, RMCs were cultured for 48 h in low- or high-glucose medium, and at the end of the treatment period, the cells were trypsinized and washed twice with ice-cold PBS and counted in a hemocytometer chamber. The cells were then lysed to measure the total protein content by the Bradford method. The total amount of protein/cell number ratio was expressed as μg/10^5^ cells and used as the hypertrophy index.

### Protein isolation

Collected cell pellets were resuspended and briefly sonicated in a cell lysis buffer (Biyuntian Biotechnology Co., Shanghai, CN) containing 1% protease inhibitor cocktail (Biyuntian Biotechnology Co., Shanghai, CN). Cell lysate was spun at 10 000 rpm for 10 min at 4°C, and the resulting supernatant was stored at -80°C. Protein concentration was determined using the BCA protein assay (Biyuntian Biotechnology Co., Shanghai, CN).

### Western blot analysis

Proteins were subjected to 8–12% SDS-PAGE and then transferred to polyvinylidene difluoride membranes (Merk Millipore, Billerica, MA, USA). Membranes were initially blocked (TBS, 0.1% Tween 20 and 5% nonfat dry milk) for 1 h. The membranes were then probed in blocking buffer containing either anti-PGC-1α or anti-DRP1 antibody overnight at 4°C, and then incubated with the appropriate horseradish peroxidase-conjugated secondary antibody. Immunoreactions were visualized with Amersham ECL Plus Western Blotting Detection Reagents (GE Healthcare). The resulting bands were quantified by densitometry.

### Data analysis

The data are expressed as mean ± SD. Comparison among more than two groups was performed by one-way analysis of variance followed by post-hoc analysis with the unpaired t-test to evaluate the significance of the differences between the two groups. Statistical significance was assumed when P < 0.05.

## Results

### PGC-1α expression is decreased in the kidneys of diabetic rats

**Fig 1:**
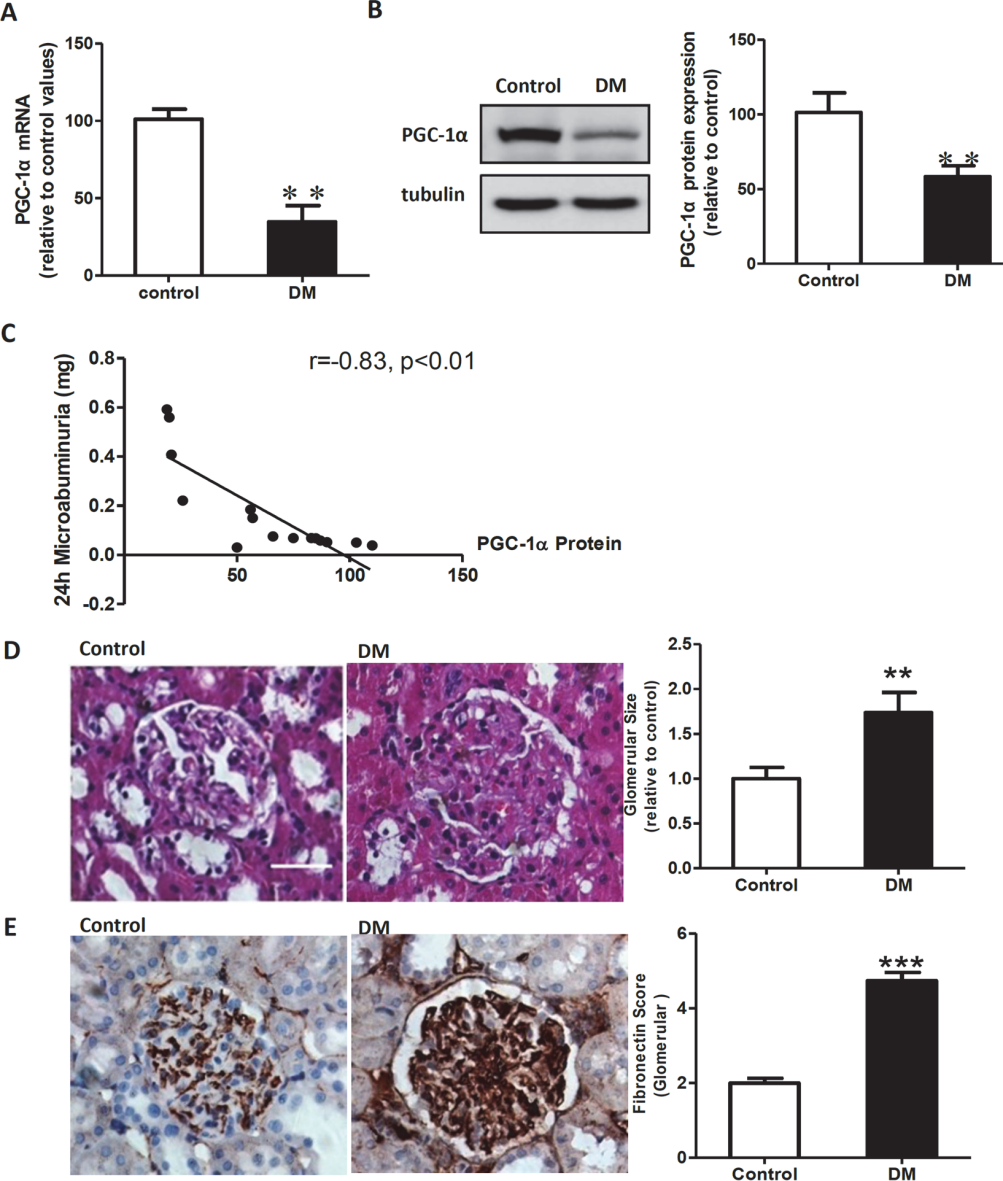
PGC-1α expression was decreased in the kidney of diabetic rats and correlated with renal lesions. A: Real-time RT-PCR assay for PGC-1α mRNA relative to the control values. B: Western blotting analysis of PGC-1α expression in the control rats (control) and diabetic rats (DM). Equal protein loading was confirmed by staining with the tubulin antibody. C: Result of the correlation analysis between PGC-1α protein expression and 24-h microalbuminuria level. D: Glomerular H&E staining in the control and diabetes groups. Glomerular hypertrophy was obvious in the diabetes group. Original magnification: ×400. E: Immunohistochemical detection of fibronectin (FN) in glomeruli from control and diabetic rats. Data are expressed as the mean ± SD values from 5–10 rats per group, and the experiments were repeated independently at least 3 times with similar results (**P < 0.01 vs. the control; ***P < 0.001 vs. the control). Scale bar: 25 μm.

The expression of PGC-1α in the renal cortex was significantly decreased in the diabetic rats at the level of both mRNA and protein; moreover, the level of proteinuria was also significantly higher in the diabetic rats compared with the control rats (Fig 1A and 1B and Table 1; P < 0.01). There was significant negative correlation between the expression of PGC-1α and the level of proteinuria (Fig 1C). In addition, pathological changes affecting the kidney were observed in diabetic rats, such as increased kidney/body weight ratio, increased glomerular volume, and increased glomerular fibronectin (FN) levels, which indicated a significant increase in the amount of extracellular matrix (Table 1 and Fig 1D and 1E; P < 0.01). Additionally, the levels of blood glucose, triglyceride, and cholesterol were significantly increased in the diabetic group compared with the control group (Table 1; P < 0.01).

**Table 1:** Physiological and biochemical data for the experimental groups (mean ± SD).

	Control	Diabetes
Number of cases	10	5
Body weight (g)	433.2 ± 29	393.3 ± 31*
Kidney weight (g)	3.16 ± 0.24	4.95 ± 0.63***
Kidney weight/Body weight (g/kg)	3.76 ± 0.12	6.29 ± 0.21***
Blood glucose (mmol/L)	6.8 ± 0.43	22.3 ± 0.95***
Total cholesterol (mmol/L)	1.27 ± 0.17	3.88 ± 1.16***
Triglycerides (mmol/L)	0.52 ± 0.19	0.94 ± 0.44*
Plasma creatinine (μmol/L)	18.7 ± 2.9	17.0 ± 1.0
24-h MA (mg)	0.07 ± 0.015	0.39 ± 0.16***

Data represent mean ± SD, MA: microalbuminuria;

*P < 0.05,

**P < 0.01,

***P < 0.001 vs. the control group.

### Decreased PGC-1α expression is associated with a hyperglycemia-induced increase in mitochondrial fragmentation and ROS generation

**Fig 2:**
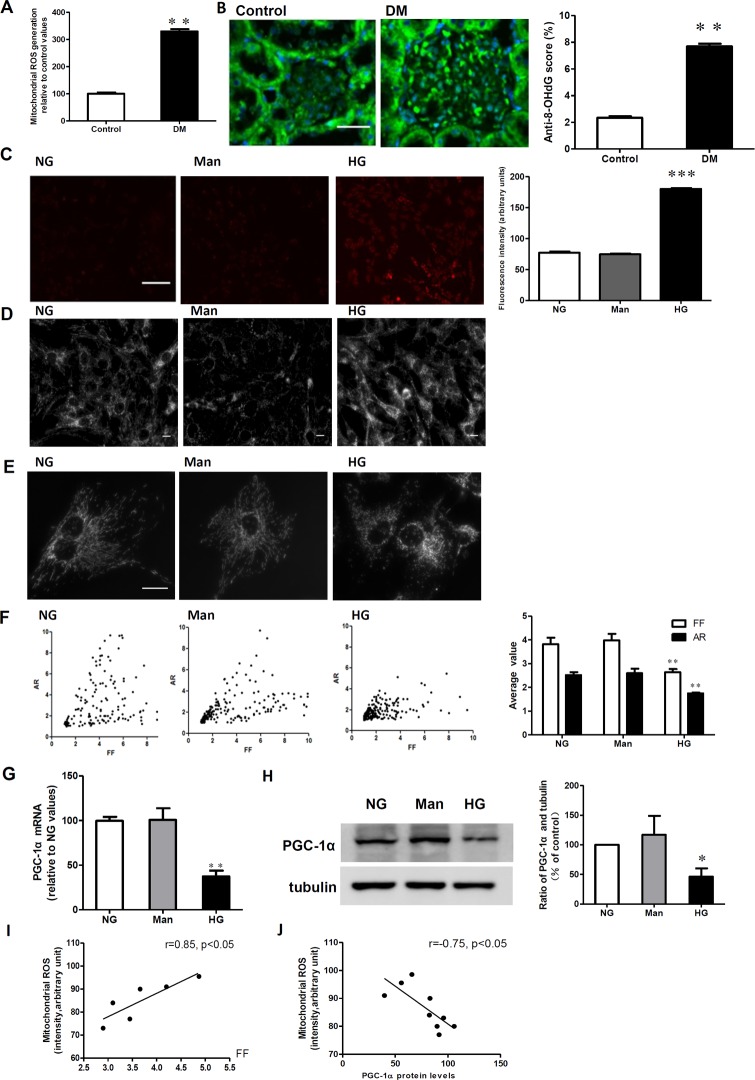
Decreased PGC-1α expression is associated with a hyperglycemia-induced increase in mitochondrial fragmentation and ROS generation. A: Kidney mitochondrial ROS production in the control and diabetic rats (DM). ROS production was identified by the fluorescent probe Mitochondrial ROS. B: Immunofluorescent micrographs of 8-hydroxy-2-deoxyguanosine (8-OHdG) in the control and diabetic rats (DM). (Original magnification: ×400). Scale bar: 25 μm. C: Intracellular ROS production was determined by the mitochondrial fluorescent probe in RMCs exposed to normal glucose (NG), mannitol (Man), and high glucose (HG). Scale bar: 100 μm. D, E and F: Mitochondrial morphology was determined using the MitoTracker probe in RMCs exposed to NG, HG, and Man. D: Mitochondrial morphology at x200. Quantitative analysis of mitochondrial morphology was conducted using a computer-assisted morphometric analysis application for the calculation of form factor (FF) and aspect ratio (AR) values. Scale bar: 10 μm. G: Real-time RT-PCR assay for PGC-1α mRNA relative to the NG values. H: Western blotting analysis of PGC-1α expression in NG, HG, and Man. I: A correlation analysis between ROS generation and the mitochondrial morphology parameter, FF. J: A correlation analysis between ROS generation and PGC-1α expression. Data are expressed as the mean ± SD values for 5–10 rats per group or three cells per group, and the experiments were repeated independently at least three times with similar results (**P < 0.01 vs. control or vs. NG).

The in vivo production of mitochondrial ROS was significantly increased in the cortex of diabetic rats (Fig 2A; P < 0.01), and the level of 8-OHdG, one of the predominant forms of free radical-induced oxidative lesions [18], was also significantly higher in the glomeruli of diabetic rats compared to control rats (Fig 2B; P < 0.01). Moreover, in vitro exposure of RMCs to high levels of glucose led to increased ROS production (Fig 2C) and decreased PGC-1α expression (Fig 2G and 2H). To exclude the influence of osmolarity on ROS generation and PGC-1α expression, mannitol was used to treat the cells, but had no effect (Fig 2C). Further mitochondrial morphology analyses indicated that mesangial cells had an extensive network of mitochondria, in which the shape of individual mitochondria was long, tubular, and highly branched under normal glucose conditions (Fig 2D and 2E). In contrast, when cells were grown under high glucose conditions, the mitochondrial network of mesangial cells appeared significantly disrupted, while in the osmotic control medium, there was no significant disruption (Fig 2D and 2E). Accordingly, the average form factor (FF) and aspect ratio (AR) values, which indicate the length and branching of the mitochondria, were lower in RMCs grown in HG medium compared to those grown in NG medium (2.64 vs. 3.82 [P < 0.01] and 1.75 vs. 2.51 [P < 0.01], respectively; Fig 2F). Pearson correlation analysis indicated that the increase in ROS generation was significantly correlated with the mitochondrial morphology parameters, FF and levels of PGC-1α protein (Fig 2I).

### PGC-1α inhibited hyperglycemia-induced elevation of ROS production as well as mitochondrial fragmentation

**Fig 3:**
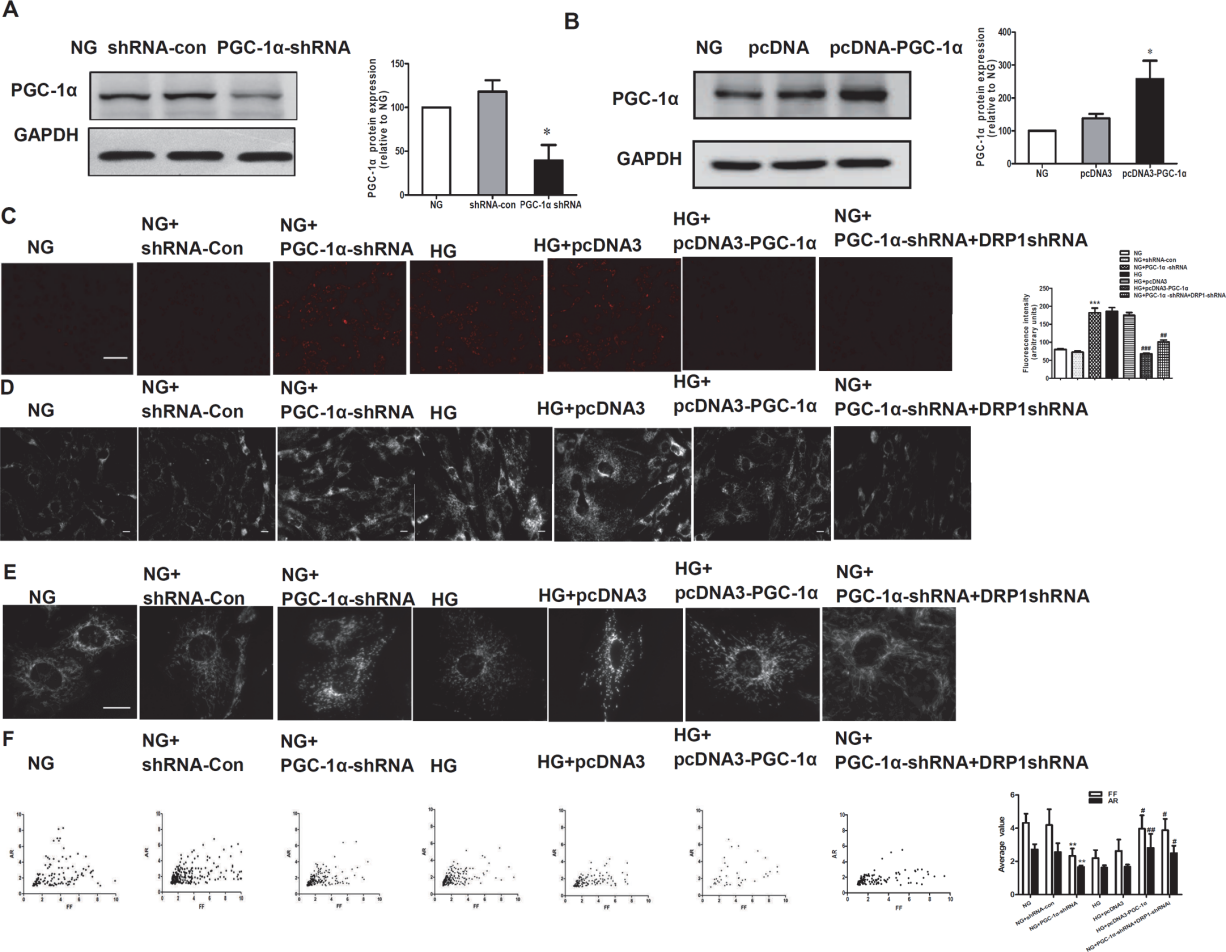
PGC-1α inhibited hyperglycemia-induced elevation of ROS production as well as mitochondrial fragmentation. A: Mesangial cells were transfected with PGC-1α short hairpin RNA (shRNA) for 48 h, and PGC-1α protein expression was detected by western blotting. PGC-1α expression was inhibited (~66% reduction) by PGC-1α shRNA. B: PGC-1α expression in transfected mesangial cells. Mesangial cells were transfected with the pcDNA3-PGC-1α (PGC-1α expression) plasmid or empty vector (pcDNA3), with untreated cells used as the control. Expression levels of PGC-1α in the indicated transfectants were analyzed by Western blotting. (C-F) ROS production, mitochondrial morphology changes, and computer-assisted morphometric analyses of mitochondrial morphology in RMCs exposed to normal glucose (NG) and high glucose (HG) conditions, RMCs transfected with PGC-1α shRNA plasmid (NG + PGC-1α shRNA) and shRNA control plasmid (NG+shRNA-con) and exposed to NG conditions, RMCs transfected with pcDNA-PGC-1α plasmid (HG + pcDNA3-PGC-1α) and empty plasmid pcDNA3 (HG + pcDNA3) and exposed to HG conditions, RMCs transfected with PGC-1α shRNA plasmid and DRP1-shRNA plasmid (NG + PGC-1α shRNA+DRP1-shRNA). ***P < 0.001, ** P < 0.01 versus NG, ## P < 0.01, # P < 0.05 versus HG. Scale bar: 10 μm.

To clarify the role of PGC-1α in the regulation of mitochondrial ROS, we first transfected RMCs with PGC-1α shRNA plasmid, or pcDNA3-PGC-1α plasmid to silence or overexpress the expression of PGC-1α (Fig 3A and 3B). As shown in Fig 3C, cells that were transfected with PGC-1α shRNA plasmid (incubated in NG medium) had significantly increased levels of ROS that were comparable to those cells under high glucose conditions (P < 0.01). Similarly, the level of mitochondrial fragmentation in RMCs transfected with PGC-1α shRNA incubated in normal glucose is comparable to that under high glucose conditions (Fig 3D and 3E). In addition, the average FF and AR values showed similar changes (Fig 3F). There were no changes in the RMCs transfected with the shRNA-con plasmid (incubated in NG medium) and in the RMCs transfected with pcDNA3 plasmid (incubated with HG medium). On the contrary, the overexpression of PGC-1α significantly decreased levels of hyperglycemia-induced mitochondrial fragmentation and subsequent ROS production (Fig 3C–3E).

### Inhibitory action of PGC-1α on mitochondrial fragmentation is via downregulation of DRP1

**Fig 4:**
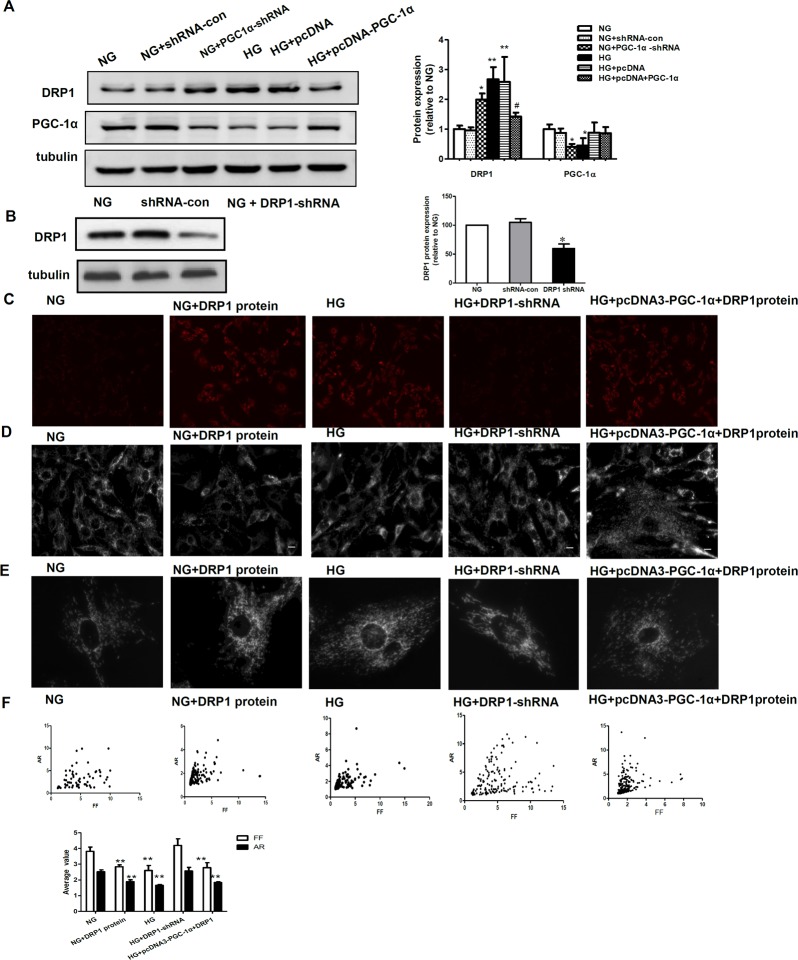
Inhibitory action of PGC-1α on mitochondrial fragmentation occurs via the downregulation of DRP1. A: Western blotting analysis of DRP-1 and PGC-1α expression in the NG, NG+shRNA-con, NG+PGC-1α shRNA, HG, HG+pcDNA3, and HG+pcDNA3-PGC-1α groups. Equal protein loading was confirmed with tubulin antibody staining. Data are presented as the mean ± SD values for three cells per group, and experiments were repeated independently at least three times (*P < 0.05 vs. NG, # P < 0.05 vs. HG). B: Mesangial cells were transfected with DRP1 short hairpin RNA (shRNA) for 48 h, and DRP1 protein expression was detected by western blotting. DRP1 expression was inhibited (~50% reduction) by DRP1 shRNA. C-F: ROS production, mitochondrial morphology changes, and computer-assisted morphometric analyses of mitochondrial morphology in RMCs exposed to normal glucose (NG), NG incubated with DRP1 protein and high glucose (HG) conditions, RMCs transfected with DRP1 shRNA to silence the expression of DRP1 under HG conditions (HG+DRP1-shRNA), and RMCs transfected with pcDNA-PGC-1α to overexpress PGC-1α and exogenous DPR1 protein under HG conditions (HG+pcDNA3-PGC-1α+DRP1). ***P < 0.001,** P < 0.01 versus NG, ## P < 0.01, # P < 0.05 versus HG.

DRP1 expression has been reported to be associated with mitochondrial fragmentation. To investigate whether DRP1 plays a role in PGC-1α-induced mitochondrial fragmentation, we measured the protein expression of DRP1 and PGC-1α in RMCs grown in NG and HG medium, and RMCs transfected with PGC-1α-shRNA and its control plasmid (shRNA-con) grown in NG medium or pcDNA-PGC-1α and its control plasmid(pcDNA3) grown in HG medium (Fig 4A). The protein expression of DRP1 was significantly higher in HG than in NG conditions, while there was no difference between the shRNA-con group grown in NG medium and the NG group, similar results were observed between RMCs transfected with PGC-1α-shRNA in NG medium and RMCs in HG medium. In addition, our results indicated that the upregulation of DRP1 induced by hyperglycemia could be inhibited by overexpression of PGC-1α.

The role of DRP1-mediated mitochondrial fragmentation in ROS generation was assessed. The exposure of RMCs to DRP1 protein in NG led to increased levels of mitochondrial fragmentation (Fig 4D–4F) and subsequent increased ROS production (Fig 4C); furthermore, after endogenous DRP1 was silenced, mitochondrial fragmentation was significantly reduced under high glucose conditions. Also, the effect of PGC-1α overexpression on mitochondrial fragmentation was abolished by the incubation of cells with DRP1 protein (Fig 4B–4F). Furthermore, PGC 1 alpha knockdown-induced mitochondrial ROS production can be prevented by Drp1 knockdown (Fig 3C–3E). These findings indicate that PGC-1α regulates mitochondrial morphology via DRP1.

### PGC-1α suppresses mesangial cell hypertrophy induced by hyperglycemia

**Fig 5:**
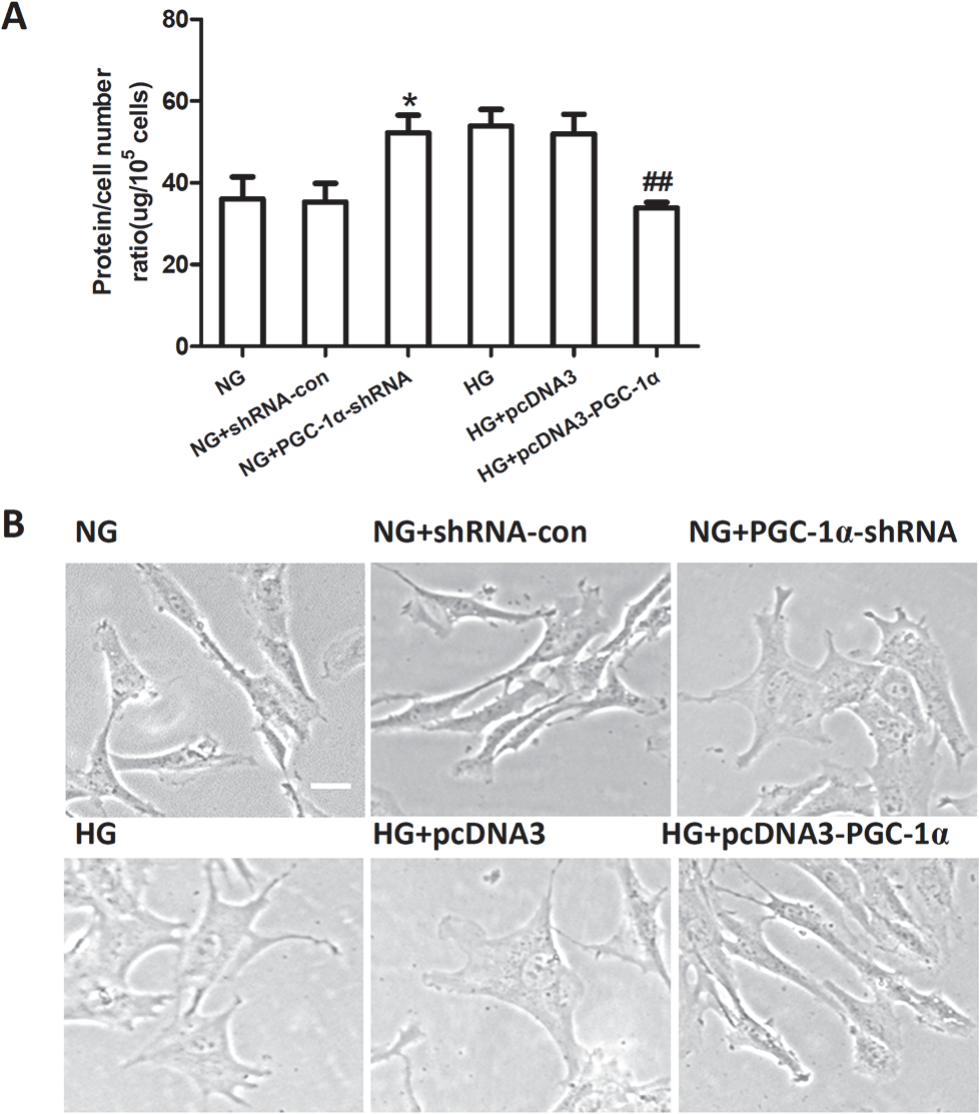
PGC-1α suppresses mesangial cell hypertrophy induced by hyperglycemia. The ratio of total amount of protein to cell number (A) and cell morphology (B) in RMCs incubated in normal glucose (NG) and high glucose (HG) conditions, RMCs transfected with PGC-1α shRNA or shRNA-con under NG conditions (NG+PGC-1α shRNA, NG+shRNA-con), and RMCs transfected with pcDNA3-PGC-1α or pcDNA3 under to HG conditions (HG+pcDNA3-PGC-1α, HG+pcDNA3). Data are represented as the mean ± SD values from three cells per group, and the experiments were repeated independently at least three times (*P < 0.05 vs. NG, ## P < 0.01 vs. HG). Scale bar: 10 μm.

The earliest morphological change in DN is mesangial cell hypertrophy, which results from an increase in protein synthesis in the absence of an increase in DNA synthesis. As shown in Fig 5, the ratio of the amount of total protein to cell number and cell morphology in RMCs with and without pcDNA3 plasmid transfection under HG conditions or in RMCs transfected with PGC-1α shRNA under NG conditions were significantly greater than NG group (P < 0.05); there were no significant differences between the NG group and the shRNA-con group (Fig 5). Moreover, we found that RMC hypertrophy promoted by hyperglycemia was ameliorated in RMCs that expressed exogenous PGC-1α (Fig 5).

## Discussion

In this study, we found that expression of PGC-1α in the kidney cortex was significantly decreased in the diabetic rats. This was accompanied by an increase in proteinuria and glomerular hypertrophy that was associated with higher levels of glomerular 8-OHdG, a biomarker of oxidative stress. Moreover, in the in vitro experiments, high levels of glucose induced the downregulation of PGC-1α, which led to increased DRP1 expression, increased mitochondrial fragmentation, and a damaged network structure. This mitochondrial dysfunction caused an increase in ROS generation and mesangial cell hypertrophy. Importantly, we also found that in vitro overexpression of PGC-1α could reverse these pathological changes.

PGC-1α expression is highly related to glucose metabolism. It has been reported that chronic hyperglycemia may reduce the expression of PGC-1α in skeletal muscle [19], isolated rat islets [20], and vascular smooth muscle cells [21]. Here, we show that PGC-1α expression is inhibited in the kidneys of STZ-diabetic rats (Fig 2). Further experiments revealed that high levels of glucose inhibit PGC-1α expression in cultured rat mesangial cells (RMCs) (Fig 2). These results indicate that high levels of glucose decrease PGC-1α expression both in vivo and in vitro, which was in accordance with the results of previous studies that showed the PGC-1α level in the kidney was significantly lower in db/db mice compared with db/m mice [18]. Furthermore, in cultured mesangial cells, high glucose (30 mmol/l D-glucose) significantly reduced the expression of PGC-1 α compared with cells incubated with 5 mmol/l glucose (low glucose) [22]. The mechanism underlying decreased PGC-1α expression levels induced by hyperglycemia is unclear. Epigenetic studies have suggested that the expression of PGC1α may be controlled at the level of DNA methylation of the PGC-1α promoter at both cytosine-guanine dinucleotide (CpG) sites and non-CpG sites. [23–25]. The increase in the level of DNA methylation at the PGC-1α promoter appears to be a mechanism shared by multiple tissue types in patients with T2DM, which reduce PGC-1α expression and mitochondrial content. However, in hepatocytes or endothelial cells, hyperglycemia induces the upregulation of PGC-1α [26,27]. The discrepant regulation of PGC-1α between different cell types might result from a tissue type-specific mechanism of gene regulation.Mitochondria are dynamic organelles that frequently change their shape, number, and intracellular distribution in response to fluctuations in metabolic demands [10]. Mitochondrial dynamics have emerged as an important process that contributes to mitochondrial dysfunction in a variety of metabolic conditions, including those associated with the diabetic milieu [28]. Our results indicated that increases in proteinuria and glomerular hypertrophy in diabetic rats are associated with increased levels of ROS production and higher levels of the oxidative stress biomarker 8-OHdG in the glomerulus; our in vitro results also suggest that mitochondrial fission is a key mediator of increased ROS production under high-glucose conditions. These findings are consistent with those reported in other studies [28–31]. Several studies have indicated previously that mitochondrial fission might be a key mediator of mitochondrial ROS generation [28–31]. In mitochondria, the majority of ROS is produced in the electron transport chain (ETC) when electrons escape from the ETC. The ETC is contained within the elaborately folded inner membrane. Thus, it is possible that a large-scale change of mitochondrial membrane caused by the fragmentation of membrane tubules in hyperglycemic conditions may change the structural organization and arrangement of ETC components within the membrane. This organizational derangement of the ETC may lead to the perturbation of ETC activity, causing the overproduction of ROS [32].

It is important to clarify the mechanism that underlies excessive mitochondrial fission in mesangial cells under hyperglycemic conditions. It is well known that changes in PGC-1α activity induce a transcriptional response, which boosts mitochondrial activity in times of energy need and attenuates it when energy demands are low [33], [34] [35]; this molecule could therefore play a role in mitochondrial fission. In our study, we found that PGC-1α inhibited changes in mitochondrial morphology and reduced ROS production that was induced by hyperglycemia in mesangial cells.

In addition, we found that the effects of PGC-1α on mitochondrial morphology changes and ROS production were brought about via the downregulation of the mitochondrial fission protein, DRP1, which is one of the important protein regulators of mitochondrial fragmentation. DRP1, a GTPase of the fission/fusion protein family, plays a role in maintaining the balance between mitochondrial fission, fusion and remodeling in response to stress and metabolic changes [36–38]. DRP1 catalyzes the process of mitochondrial fission, which is a critical mechanism for both the effects and production of ROS, and plays a central role in diabetes and diabetic complications [39]. Silencing DRP1 expression with siRNA has been reported to blunt hyperglycemia-induced changes in mitochondrial networks and ROS production [28,40]. We observed that inhibiting DRP1 expression had a similar effect; that is, it was sufficient to completely inhibit the glucose-induced ROS production and network fragmentation in mesangial cells. Thus, our findings appear to indicate that mitochondrial fission mediated by DRP1 is a major cause of mesangial cell dysfunction in the setting of hyperglycemia, which is probably a result of increased mitochondrial ROS. However, the mechanism that underlies the effects of PGC1α on the expression of DRP1 needs to be clarified.

Recent studies have indicated that mitochondrial ROS can regulate the expression of proinflammatory cytokines to induce mesangial cell proliferation, glomerular hypertrophy, and the thickening of the glomerular basement membrane under hyperglycemic conditions [41–43]. Our results suggested that PGC-1α overexpression may result in the suppression of inflammation mediated by ROS through the DRP1/mitochondrial dynamic system in DN.

In summary, we have demonstrated that PGC-1α decreased DRP1 expression, which improves mitochondrial fragmentation and network structure, and subsequently leads to a decrease in mitochondrial ROS generation. This resulted in improvements in the pathological changes found in DN. Our studies suggest that pharmacological modulation of PGC-1α protein activity might be a promising therapeutic strategy for the treatment of patients with DN.

## Supporting Information

S1 Fig Mitochondrial Morphology analysis method. Computer-assisted quantitative analyses of mitochondrial morphology. Digital images were subjected to a convolve filter through the National Institutes of Health-developed IMAGEJ software to isolate and equalize fluorescent pixels in the image. After thresholding, individual particles (mitochondria) were analyzed for circularity (4π×Area/(perimeter^2^)) and lengths of major and minor axes. From these values, we calculated form factor (FF; the reciprocal of circularity value) and aspect ratio (AR; major/minor). Both FF and AR have a minimal value of 1 when a particle is a small perfect circle and the values increase as the shape becomes elongated. Specifically, AR is a measure of mitochondrial length, and increase of FF represents increase of mitochondrial length and branching.
(TIF)
