# Supplementary figures and images for: Protective Role of PGC-1α in Diabetic Nephropathy Is Associated with the Inhibition of ROS through Mitochondrial Dynamic Remodeling
Source: PLoS One. 2015 Apr 8;10(4):e0125176. doi: 10.1371/journal.pone.0125176 (PMC4390193; doi:10.1371/journal.pone.0125176)

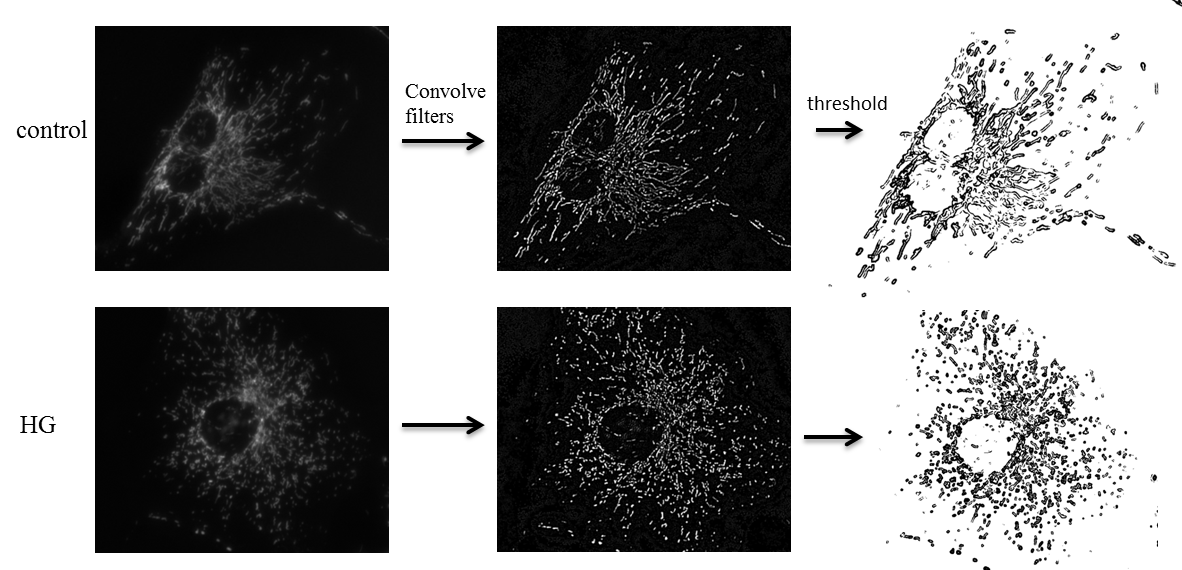

Supplement: S1 Fig — Computer-assisted quantitative analyses of mitochondrial morphology. Digital images were subjected to a convolve filter through the National Institutes of Health-developed IMAGEJ software to isolate and equalize fluorescent pixels in the image. After thresholding, individual particles (mitochondria) were analyzed for circularity (4π×Area/(perimeter2)) and lengths of major and minor axes. From these values, we calculated form factor (FF; the reciprocal of circularity value) and aspect ratio (AR; major/minor). Both FF and AR have a minimal value of 1 when a particle is a small perfect circle and the values increase as the shape becomes elongated. Specifically, AR is a measure of mitochondrial length, and increase of FF represents increase of mitochondrial length and branching. (TIF) [file pone.0125176.s001.tif]
